# Analgesic effects of different concentrations of ropivacaine in transversalis fascia plane block during laparotomy

**DOI:** 10.1186/s12871-022-01595-8

**Published:** 2022-02-26

**Authors:** Ye Tian, Yong Zhan, Ke Liu, Shaojin Bu, Yalin Tian, Chunyan Xiong, Jintao Shen

**Affiliations:** 1 Department of Anesthesiology, Pangang Group General Hospital, Panzhihua, Sichuan China; 2 Department of Anesthesiology, Fengdu People’s Hospital, No. 33, Lutang Street, Sanhe Street, 408200, Fengdu County, Chongqing, China

**Keywords:** Laparotomy, Transversalis fascia plane block, Different concentrations, Ropivacaine

## Abstract

**Objective:**

To explore the analgesic effects of different concentrations of ropivacaine in transversalis fascia plane (TFP) block during laparotomy.

**Methods:**

Ninety patients who underwent laparotomy admitted to our hospital from March 2019 to March 2020 were selected as the study subjects and were divided equally into a low concentration group, a medium concentration group, and a high concentration group according to the randomized grouping method. The low concentration group adopted 0.4% ropivacaine 40 ml, the medium concentration group was given 0.5% ropivacaine 40 ml, and the high concentration group was given 0.6% ropivacaine 40 ml. The hemodynamic indexes and the incidence of adverse reactions in the two groups were compared. The Numerical Rating Scale (NRS) was used to assess the postoperative pain in the three groups, the Bruggrmann comfort scale (BCS) was used to assess the comfort level in the three groups, and the Mini-mental State Examination (MMSE) was used to evaluate the postoperative cognitive function of the three groups of patients.

**Results:**

The mean artery pressure (MAP) and heart rate (HR) levels at T1 and T2 were significantly lower in the medium concentration group than in the other two groups (*P* < 0.05). The low concentration group had a significantly higher NRS score at T2 than the medium concentration group and the high concentration group (*P* < 0.05). A significantly higher BCS score was observed in the high concentration group than the other two groups (*P* < 0.05). There were significantly higher Ramsay scores and MMSE scores in the medium concentration group than in the low concentration and high concentration groups (*P* < 0.05). The overall incidence of adverse reactions of the high concentration group was significantly higher than that of the low concentration group (*P* < 0.05), but showed similar results with the medium concentration group.

**Conclusion:**

The medium concentration group exhibits a better analgesic effect than the low concentration group and higher safety than the high concentration group. Therefore, the use of medium concentration ropivacaine in TFP block may provide a referential basis for clinical treatment.

## Introduction

Gastric diseases, duodenal diseases, and diseases of the hepatobiliary system all require laparotomy [1, 2]. Clinical experience has revealed a close correlation of surgical outcome and quality of awakening with postoperative pain, and as noted in some studies, postoperative pain is a major factor for complications [3, 4]. In addition, postoperative pain predisposes to systemic stress and induces inflammation, which impairs body function [5, 6]. Therefore, postoperative analgesia conforms to the concept of comfort care and facilitates the prognosis of patients. In previous studies, 40 ml of ropivacaine at a concentration of 0.4% has been mostly applied for transversalis fascia plane (TFP) block in open surgeries, with significant analgesic effect. Nevertheless, the impact of variation in the concentration of ropivacaine on analgesic effect and duration of analgesia requires further investigation [7–9]. Accordingly, to further investigate the variability of the analgesic effect of different concentrations of ropivacaine in TFP block during laparotomy, 90 patients who underwent open surgeries admitted to our hospital from March 2019 to March 2020 were selected as study subjects, and the results are summarized as follows.

## Materials and methods

### General information

It was a prospective randomized controlled study to explore the analgesic effects of different concentrations of ropivacaine (high, medium and low) in TFP block during laparotomy. For a one-way ANOVA comparing 3 groups, calculate the sample size needed in each group to obtain a power of 0.80, when the effect size is large (0.4) and a significance level of 0.05 is employed. Calculated by “pwr” package of R software, the minimum sample size of each group was 21. Ninety patients who underwent laparotomy admitted to our hospital from March 2019 to March 2020 were selected. All enrollments were equally randomized the ratio of sample size of the three groups was 1:1:1 into a low concentration group, a medium concentration group, and a high concentration group according to the random grouping method.

### Inclusion and exclusion criteria

Inclusion criteria: ①Patients aged > 18 years; ②Grade I-III according to the American Society of Anesthesiologists (ASA) classification standard; ③Patients with a clear clinical diagnosis and scheduled laparotomy; ④Patients with a Montreal Cognitive Assessment (MoCA) score [10] ≥26 points; ⑤The study was in accordance with the Declaration of Helsinki [11].

Exclusion criteria: ①Patients with a history of drug allergies; ②Patients with current use of psychotropic drugs such as antidepressants and antianxietics; ③Patients with severe hearing and visual impairment; ④Patients with abnormal preoperative blood picture and hemagglutination; ⑤Patients with skin breakdown infection at the puncture site.

### Methods

Patients were fasted for 8 h and abstained from water for 4 h before surgery. All patients received standardized general anesthesia technique and perioperative analgesia as follows. The preoperative examination was performed to verify patients’ information and conditions. Vital signs such as pulse oximetry, blood pressure, and heart rate of the patients were monitored after entering the operating room. A bilateral TFP block was performed under ultrasound guidance with patients induced general anesthesia. After routine sterilization of the puncture site, the ultrasound probe was placed between the iliac crest and the rib cage to scan the internal oblique abdominal muscle, external oblique abdominal muscle, and transverse abdominal muscle. The posterior quadratus lumbosum muscle block was used, and the injection point was 1 cm outside the probe. Each group was added with 1:200,000 epinephrine. Then 0.4, 0.5 and 0.6% ropivacaine (3 mg/kg) were injected respectively in different groups. The diffusion of ropivacaine on the posterior side of quadratus lumborum muscle and in the space between thoracolumbar fascia under ultrasound indicated that the block was successful. Contralateral quadratus lumborus block was performed by the same senior anesthesiologist who was unaware of the grouping. The block was considered effective when there was no spindle-shaped echogenic signal in the transverse fascia. After successful block, anesthesia induction was conducted by midazolam 0.10–0.20 mg/kg, sufentanil 0.5–1.0 μg/kg, cis-atracurium 0.15–0.20 mg/kg, propofol 1.5–3.5 mg/kg. Anesthesia was maintained by all intravenous anesthesia: Remifentanil 0.2–0.4 μg/ (kg·min) + propofol 5–8 mg/ (kg·h) + cefatracurium 0.1–0.15 mg/ (kg·h) maintained BIS 40–60, blood pressure and heart rate within 20% of the fluctuation range of preoperative basic values. All patients underwent endotracheal intubation and general anesthesia and intraoperative management by the same responsible anesthesiologist who was unaware of the patient grouping.

The low concentration group was treated with 0.4% ropivacaine, the medium concentration group with 0.5% ropivacaine, and the high concentration group with 0.6% ropivacaine. Contralateral quadratus lumborus block was performed with a dose of 20 ml on each side for all three groups.

All procedures were performed by the same group of surgeons.

### Observation indexes

The hemodynamic indexes of the three groups of patients before surgery, 30 min during surgery, and after surgery were observed and compared, including heart rate (HR) and mean artery pressure (MAP).

The pain level of the three groups of patients at 12 h, 24 h, and 48 h postoperatively was evaluated by referring to the Numerical Rating Scale (NRS) [12], which has a total score of 10 points, rating the pain from 0 (no pain) to 10 (worst pain). A higher score indicates a greater pain of the patients.

The Bruggrmann comfort scale (BCS) [13] was used to evaluate the comfort level of patients in the three groups, in which 0 points were considered as continuous pain, 1 point as no pain at rest and severe pain during deep breathing or coughing, 2 points as no pain when lying at rest and slight pain during deep breathing or coughing, 3 points as no pain during deep breathing, and 4 points as no pain during coughing.

The cognitive function of patients was evaluated with reference to the Mini-mental State Examination (MMSE) [14], with a total score of 30 points. Higher scores represent a better cognitive function of patients.

The incidence of adverse reactions was compared among the three groups, including dizziness, tinnitus, nausea and vomiting, and hypotension.

### Statistical analyses

The data processing software selected for this study was SPSS 20.0 and GraphPad Prism 7 (GraphPad Software, San Diego, USA) was used to plot the graphics. The categorical data involved was expressed as (n, %) and analysed by chi-square test; The measursement data was expressed as ($\overline{x}$ ±s) and analysed by One-way ANOVA and post hoc test. The differences were statistically significant when *P* < 0.05.

## Results

### Comparison of clinical information

The three groups of patients had no significant differences in demographic data, such as age, height, BMI, disease type, and place of residence (*P* > 0.05), as shown in Table 1.

**Table 1:** Comparison of demographic data of the three groups

	Low concentration group(*n* = 30)	Medium concentration group(*n* = 30)	High concentration group(*n* = 30)	χ^2^ or t	P
Age (year)				0.282	0.780
	40.37 ± 2.32	40.11 ± 2.45	40.23 ± 2.38		
Height (cm)				0.142	0.887
	1.67 ± 0.38	1.65 ± 0.35	1.66 ± 0.36		
BMI (kg/m^2^)				0.141	0.887
	27.31 ± 2.33	27.22 ± 2.45	27.35 ± 2.27		
Type of disease					
Gastric cancer	18	19	17	0.139	0.727
Colon cancer	8	9	9	0.055	0.849
Abdominal masses	4	2	4	0.494	0.593
Place of residence				0.317	0.853
Urban	20 (66.67)	21 (70.00)	22 (73.33)		
Rural	10 (33.33)	9 (30.00)	8 (26.67)		

### Comparison of hemodynamic indexes

**Fig. 1:**
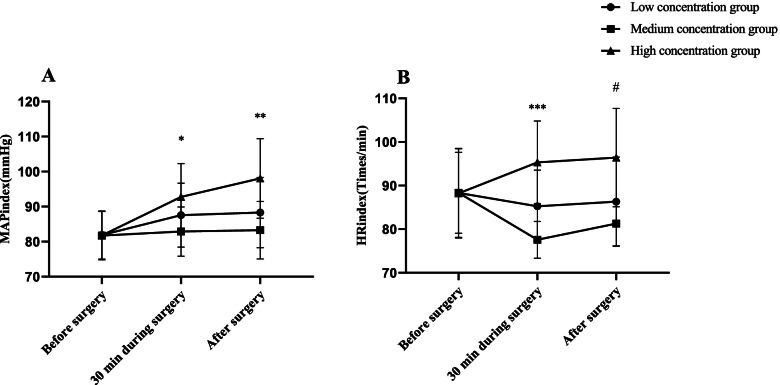
Comparison of hemodynamic indexes in the three groups ($\overline{x}$ ±s). Note: **A** shows the comparison of MAP levels at different time points in the three groups. The abscissa is the T0, T1, and T2 time points, and the ordinate is the MAP level, mmHg. The MAP levels at T0, T1, and T2 for patients in the low concentration group were (81.88 ± 6.88) mmHg, (87.55 ± 9.12) mmHg and (88.32 ± 10.04) mmHg, respectively. The MAP levels at T0, T1, and T2 of patients in the medium concentration group were (81.72 ± 6.89) mmHg, (82.88 ± 7.02) mmHg and (83.27 ± 8.21) mmHg, respectively. The MAP levels at T0, T1, and T2 in the high concentration group of patients were (81.75 ± 6.91) mmHg, (92.77 ± 9.51) mmHg and (98.05 ± 11.35) mmHg, respectively. * indicates a significant difference in MAP levels at T1 among the three groups (t = 2.992, *P* < 0.05). ** indicates a significant difference in MAP levels between the three groups at T2 (t = 3.801, *P* < 0.05). **B** shows the comparison of HR levels in the three groups at different time points. The abscissa is the T0, T1, and T2 time points, and the ordinate is the HR level, times/min. The HR levels at T0, T1, and T2 for patients in the low concentration group were (88.33 ± 10.19) beats/min, (85.24 ± 8.31) beats/min and (86.31 ± 10.24) beats/min, respectively. The HR levels at T0, T1, and T2 of patients in the medium concentration group were (88.34 ± 9.27) beats/min, (77.57 ± 4.21) beats/min and (80.33 ± 9.47) beats/min, respectively. The HR levels at T0, T1 and T2 in patients of the high concentration group were (86.31 ± 10.24) beats/min, (81.27 ± 5.09) beats/min and (96.42 ± 11.27) beats/min, respectively. *** indicates a significant difference in the HR levels at T1 among the three groups (t = 3.269, *P* < 0.05). # indicates a significant difference in the HR levels at T2 among the three groups (t = 3.991, *P* < 0.05)

The MAP and HR levels in the medium concentration group before surgery and 30 min during surgery were significantly lower than those in the low and high concentration groups (*P* < 0.05), indicating more more stable MAP and HR. As shown in Fig. 1.

### Comparison of pain scores

The NRS scores 24 h postoperatively in the low concentration group were both significantly higher than those in the medium and high concentration groups (*P* < 0.05), as shown in Table 2.

**Table 2:** Comparison of pain scores in the three groups at different time points ($\overline{x}$ ±s)

Groups	Timepoints	NRS scores
Low concentration group	12 h postoperatively	8.11 ± 1.25
	24 h postoperatively	7.88 ± 1.01
	48 h postoperatively	5.33 ± 0.87^*#^
Medium concentration group	12 h postoperatively	8.09 ± 1.23
	24 h postoperatively	6.47 ± 0.85
	48 h postoperatively	4.05 ± 0.51^*#^
High concentration group	12 h postoperatively	8.13 ± 1.27
	24 h postoperatively	5.93 ± 0.72
	48 h postoperatively	3.17 ± 0.25^*#^

*Note*: * represents *P* < 0.05 in the comparison between this group at T2 and T0, # represents *P* < 0.05 in the comparison between the medium concentration group at T2 and the low concentration group and the high concentration group

### Comparison of BCS scores

**Fig. 2:**
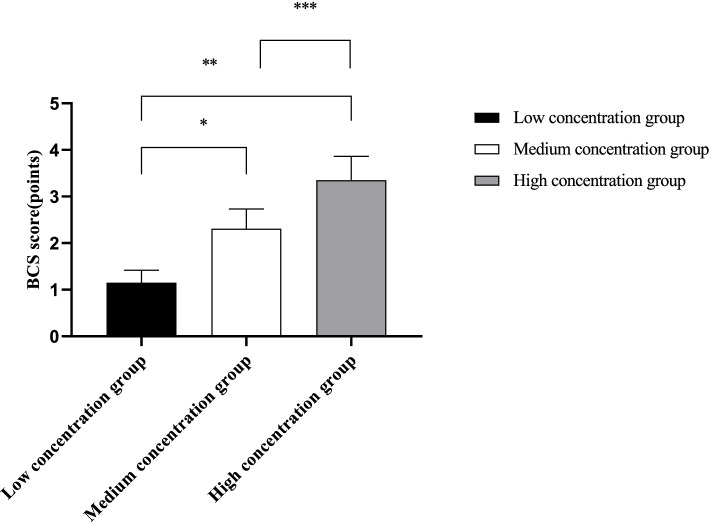
Comparison of BCS scores in the three groups ($\overline{x}$ ±s). Note: The abscissa indicates the low concentration group, medium concentration group, and high concentration group, respectively, and the ordinate indicates the BCS score, in points. The BCS scores of the low, medium, and high concentration groups were (1.15 ± 0.27), (2.31 ± 0.42), and (3.35 ± 0.51), respectively. * indicates a significant difference in BCS scores between the low and medium concentration groups (t = 12.725, *P* < 0.001). ** indicates a significant difference in BCS scores between the low concentration group and the high concentration group (t = 20.881, *P* < 0.001). *** indicates a significant difference in BCS scores between the medium concentration group and the high concentration group (t = 8.622, *P* < 0.001)

The high concentration group showed significantly higher BCS scores than the low and medium concentration groups (*P* < 0.05), as detailed in Fig. 2.

### Comparison of MMSE scores

The MMSE scores in the medium concentration group were significantly higher than those in the low concentration and high concentration groups (*P* < 0.05), as shown in Table 3.

**Table 3:** Comparison of MMSE scores of the three groups ($\overline{x}$ ±s)

Groups	n	Postoperatively
Low concentration group	30	23.31 ± 1.45
Medium concentration group	30	28.15 ± 1.23^*#^
High concentration group	30	20.27 ± 1.14

*Note*: * represents *P* < 0.05 in the comparison between this group and the low concentration group; # represents *P* < 0.05 in the comparison between this group and the high concentration group

### Comparison of the incidence of adverse reactions

The total incidence of adverse reactions in the high concentration group was significantly higher than that in the low dose group (*P* < 0.05), and differences were absent between the high concentration group and the medium concentration group. However, the number of adverse reactions cases in the high concentration was higher than that in the medium concentration group. See Table 4.

**Table 4:** Comparison of the incidence of adverse reactions between the three groups [n(%)]

Groups	n	Dizziness	Tinnitus	Nausea and vomiting	Hypotension	Total incidence
Low concentration group	30	1 (3.33%)	0 (0.00%)	1 (3.33%)	1 (3.33%)	3 (10.00%)
Medium concentration group	30	1 (3.33%)	1 (3.33%)	2 (6.67%)	1 (3.33%)	5 (16.67%)
High concentration group	30	3 (10.00%)	2 (6.67%)	3 (10.00%)	2 (6.67%)	10 (33.33%)^*^

*Note*: * represents *P* < 0.05 in the comparison between this group and the low concentration group

## Discussion

Diseases such as colon cancer, abdominal masses, and gastric cancer mostly require open surgery [15], and the presence of postoperative pain after anesthesia will severely compromise patients’ prognosis, especially in elder patients, which necessitates effective analgesic measures [16, 17]. Clinical experience has revealed intolerance to intrathecal anesthesia in some patients, which prevents its extensive use in practice. TFP block is by injecting anesthetics into the patient’s abdominal transverse fascia at the lateral cutaneous branch of the spinal nerve to block nerve impulse conduction, thereby mitigating pain in muscles, skin, and abdominal wall [18, 19]. Ropivacaine has little damage to the human heart and nerves, so it not only has good anesthesia effect, but also maintains certain tension in the lower limbs during anesthesia, which can enhance the peripheral vascular resistance, promote the vasoconstriction, so as to stabilize the blood circulation and dynamics, and benefit the venous reflux [20]. However, the effect of anesthetic drugs is related to drug concentration. With the increase of the dosage of anesthetic drugs, the risk of adverse reactions increases along with the increase of anesthetic effects. Therefore, an appropriate anesthetic concentration for general anesthesia induction can ensure an uneventful operation and improve the quality of life of patients.

In this study, more stable levels of MAP and HR in the medium concentration group than those of the low and high concentration groups at T1 and T2 indicate that the medium concentration of ropivacaine presents a lower effect on the hemodynamics of patients. Ropivacaine is a new type of long-acting amide local anesthetic with low toxicity and rapid onset of action, which can promote rapid physical recovery of patients, and is therefore widely applied in abdominal surgery. Ropivacaine has been reported to produce reversible blockade of impulse conduction along nerve fibers by inhibiting nerve cell sodium channels and blocking the flow of sodium ions into the nerve fiber cell membrane, and it also has little impact on the motor nerve in patients [21]. Previous study has proved that ropivacaine anesthesia requires a threshold volume and concentration, and concentration primarily determines motor block. When used in combination with continuous blockade, the above-threshold dose of ropivacaine does not significantly prolong the time to initial blockade, but may impair patient satisfaction. However, there was no consistency in drug concentration [22]. In the current study, both NRS and BCS scores at T2 were significantly higher in the high concentration group than in the low concentration group and the high concentration group, indicating that, the higher the concentration of anesthesia the less painful and the more comfortable the patients were. Ropivacaine is a new local anesthetic that inhibits neuronal excitement and conduction by inhibiting neuronal sodium channels. It has strong analgesic effect and can produce nerve block effect at low concentration. It is a common anesthetic used in nerve block anesthesia because of its long lasting effect and low inhibitory activity in central nervous system. There are no consistent conclusions about the analgesic effects of different concentrations [23]. However, it was found that some patients were predisposed to various adverse reactions after the use of ropivacaine, such as dizziness, tinnitus, nausea and vomiting, and the higher the concentration, the higher the risk of adverse reactions [24]. The results of this study showed that the number of cases with adverse reactions in the high concentration group was higher than the other two groups, but significant differences were only observed in the comparison with the low concentration group but absent in the comparison with the medium concentration group, which is consistent with the results of Kao Hao-Wen et al. [25], who stated in their article that the incidence of drug side effects of high concentration of ropivacaine was significantly higher than that in low concentration, which was 6.67% (*p* < 0.05). It fully illustrates a higher safety profile of medium concentration compared to high concentration ropivacaine.

## Conclusion

To sum up, the medium concentration group exhibits a better analgesic effect than the low concentration group and higher safety than the high concentration group. Therefore, the use of medium concentration ropivacaine in TFP block may provide a referential basis for clinical treatment.

## Data Availability

The datasets used and analysed during the current study are available from the corresponding author on reasonable request.
